# Phenotyping of chronic lung allograft dysfunction in the ScanCLAD study

**DOI:** 10.1183/23120541.00084-2026

**Published:** 2026-08-17

**Authors:** Jesper M. Magnusson, Peter Raivio, Inga Leuckfeld, Johan Svahn, Erik Holmberg, Thomas Kromann-Lund, Göran Dellgren

**Affiliations:** 1 Department of Pulmonology, Sahlgrenska University Hospital, University of Gothenburg, Gothenburg, Sweden; 2 Transplant Institute Gothenburg, Gothenburg, Sweden; 3 Department of Cardiac Surgery, Helsinki University Hospital, University of Helsinki, Helsinki, Finland; 4 Department of Respiratory Medicine, Oslo University Hospital, Oslo, Norway; 5 Department of Pulmonology and Allergology, Skåne University Hospital, Lund, Sweden; 6 Department of Oncology, Institute of Clinical Sciences, University of Gothenburg, Gothenburg, Sweden; 7 Section for Lung Transplantation, Department of Cardiology, Rigshospitalet, Copenhagen University Hospital, Copenhagen, Denmark; 8 Department of Cardiothoracic Surgery, Sahlgrenska University Hospital, University of Gothenburg, Gothenburg, Sweden

## Abstract

Chronic lung allograft dysfunction (CLAD) remains the primary cause of long-term death or graft-failure after lung transplantation. The ScanCLAD trial demonstrated that tacrolimus-based immunosuppression significantly reduced CLAD incidence compared to ciclosporin (13% *versus* 39% at 3 years). However, CLAD phenotyping was not part of the original protocol. We report the first comprehensive phenotype analysis from a prospective randomised controlled trial in lung transplantation, employing a structured consensus process to classify CLAD phenotypes and assess their stability over time.

## Abstract 2

**First phenotype analysis from a randomised lung transplant trial: 16% of CLAD cases were unclassifiable and 34% showed a phenotype drift, highlighting the urgent need to refine the current classification criteria** https://bit.ly/41ACqyW

*To the Editor:*

Chronic lung allograft dysfunction (CLAD) remains the primary cause of long-term death or graft-failure after lung transplantation. The ScanCLAD trial demonstrated that tacrolimus-based immunosuppression significantly reduced CLAD incidence compared to ciclosporin (13% *versus* 39% at 3 years). However, CLAD phenotyping was not part of the original protocol. We report the first comprehensive phenotype analysis from a prospective randomised controlled trial in lung transplantation, employing a structured consensus process to classify CLAD phenotypes and assess their stability over time.

The ScanCLAD study randomised 249 lung transplant recipients across five Scandinavian centres to tacrolimus or ciclosporin-based immunosuppression. CLAD was defined as persistent ≥20% forced expiratory volume in 1 s (FEV_1_) decline from baseline, confirmed by repeat spirometry after three weeks, with adjudication by an independent committee blinded to treatment allocation [1]. Among 64 patients who developed CLAD within three years, we performed *post hoc* phenotyping according to 2019 International Society for Heart and Lung Transplantation (ISHLT) criteria [2]: bronchiolitis obliterans syndrome (BOS); restrictive allograft syndrome (RAS); mixed; undefined; and a fifth category – unclassifiable – for presentations not covered by current guidelines.

Six raters (five principal investigators and the sponsor) independently classified all cases based on standardised data packages containing spirometry, total lung capacity (TLC) when available, and high-resolution computed tomography. Inter-rater agreement was assessed using Conger's kappa [3], Krippendorff's alpha, and observed pairwise agreement, with uncertainty quantified *via* patient-clustered bootstrap (1000 replicates) [4]. We used both methods to demonstrate robustness of agreement across different statistical frameworks. Both yielded identical point estimates (0.69) and confidence intervals, indicating consistent inter-rater reliability regardless of statistical approach. Krippendorff's alpha is technically preferred for multiple raters, while Conger's kappa facilitates comparison with prior two-rater studies in the CLAD phenotyping literature.

Following independent rating, a modified Delphi consensus process was conducted. Cases were presented in standardised format without suggested phenotypes. Anonymous voting occurred *via* an online platform, with consensus defined as ≥5 out of 6 votes (>80% agreement). Cases without consensus underwent open discussion followed by repeat voting. Obstruction was defined as FEV_1_/FVC (forced vital capacity) <0.7. Restriction required TLC decline ≥10% from baseline; when TLC was unavailable, FVC decline ≥20% with FEV_1_/FVC ≥0.7 or increased from baseline was used [5].

Phenotype instability is a well-known phenomenon [6]. Phenotyping at end-of-study (EOS) (death, re-transplantation, or three-year follow-up) was performed for cases with >3 months between CLAD diagnosis and EOS with available spirometry; change between these two timepoints was termed phenotype drift.

Independent classification of the 64 patients with CLAD at three years yielded observed pairwise agreement of 0.80 (95% CI 0.66–0.94) and Conger's kappa of 0.69 (95% CI 0.49–0.90), indicating substantial agreement beyond chance. Krippendorff's alpha was 0.69 (95% CI 0.49–0.90). Category-specific agreement was very high: BOS 1.00, RAS 0.99, mixed 0.98, undefined 0.99, and unclassifiable 1.00. This compares favourably with the only prior inter-rater study, which reported 73% agreement and kappa of 0.291 in 48 CLAD patients [7].

Initial consensus (first vote) was achieved in 60 of 64 (94%) cases at CLAD diagnosis. Seven cases required discussion and repeat voting, all occurring within the first third of sessions. Complete consensus (6 of 6 votes) was ultimately achieved in 86% of cases.

In the ciclosporin arm (n=48), phenotypes were: BOS 26 (54%), RAS 6 (13%), mixed 3 (6%), undefined 8 (17%), and unclassifiable 5 (10%). In the tacrolimus arm (n=16): BOS 8 (50%), RAS 1 (6%), mixed 1 (6%), undefined 1 (6%), and unclassifiable 5 (31%) (figure 1). Despite higher unclassifiable proportion in the tacrolimus arm, the small sample size precludes statistical comparison between treatment groups.

**FIGURE 1:**
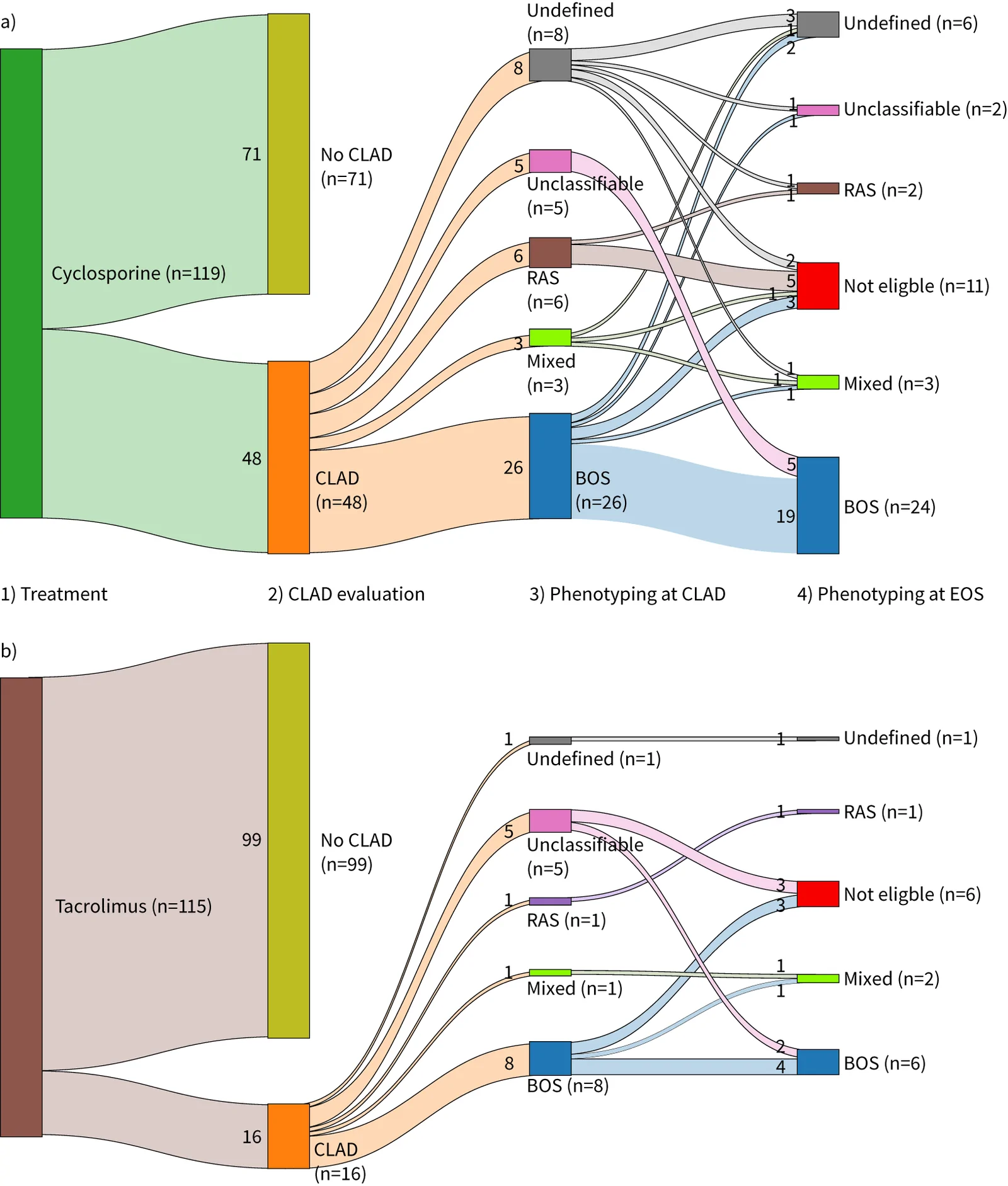
Sankey diagram showing patient flow from treatment allocation through chronic lung allograft dysfunction (CLAD) development, initial phenotyping, and phenotype evolution at end-of-study (EOS). a) The cyclosporine arm (n=119 randomised and available for CLAD evaluation); b) the tacrolimus arm (n=115 randomised and available for CLAD evaluation). Flow widths are proportional to patient numbers. 1) Treatment shows total patients randomised to each arm. 2) CLAD evaluation at 3 years shows patients who developed CLAD (orange) *versus* those who remained CLAD-free (yellow/green). 3) Phenotyping at CLAD shows distribution across five phenotype categories. 4) Phenotyping at EOS shows final phenotype classification and patients not eligible for re-evaluation (red). Among 64 CLAD patients (48 cyclosporine, 16 tacrolimus), phenotype distributions at diagnosis were similar between arms. Bronchiolitis obliterans syndrome (BOS) predominated in both (cyclosporine 26 of 48 (54%), tacrolimus eight of 16 (50%)), while unclassifiable cases represented five of 48 (10%) in the cyclosporine and five of 16 (31%) in the tacrolimus arms. Of 47 patients eligible for EOS evaluation (37 cyclosporine, 10 tacrolimus), phenotype drift occurred in 16 (34%). Most notably, all re-evaluated unclassifiable patients transitioned to other phenotypes (pink flows), indicating early CLAD presenting before spirometric obstruction becomes apparent. BOS remained the most stable phenotype (blue flows), while several patients developed new radiological opacities during follow-up, transitioning from non-opacity to opacity-associated phenotypes (flows from blue to gray/brown bands). Not eligible patients (n=17; 11 cyclosporine, six tacrolimus) had <3 months follow-up after CLAD diagnosis due to death, re-transplantation or receiving the diagnosis close to EOS. RAS: restrictive allograft syndrome; Mixed: combined obstructive and restrictive features with opacities; Undefined: presentations meeting CLAD criteria but not fitting BOS, RAS, or Mixed definitions; Unclassifiable: presentations not covered by current International Society for Heart and Lung Transplantation consensus criteria.

TLC was unavailable in 10 of 64 (16%) cases at CLAD diagnosis, necessitating FVC-based restriction criteria. Eight of 10 unclassifiable cases presented with isolated FEV_1_ decline without spirometric obstruction (FEV_1_/FVC >0.7), restriction, or radiological opacities. Two cases had restriction without obstruction or opacities.

Among 47 patients eligible for EOS evaluation (37 ciclosporin, 10 tacrolimus), 16 (34%, 95% CI 21–49%) demonstrated phenotype drift (figure 1, right-most column). TLC was unavailable in 20 of 47 (43%) cases at EOS. The most common drift pattern involved unclassifiable cases developing BOS: all seven re-evaluated unclassifiable patients (100%) transitioned to BOS at EOS (shown by pink flows in figure 1), characterised by development of spirometric obstruction that was absent at CLAD diagnosis. Among patients classified as BOS at diagnosis, the majority remained stable (shown by thick blue flows). Several patients developed new radiological opacities during follow-up, with transitions visible between phenotype categories in figure 1.

This study provides the first phenotyping analysis from a prospective randomised trial in lung transplantation. Despite substantial inter-rater agreement (kappa 0.69), higher than previously reported, 16% of cases were unclassifiable at CLAD diagnosis and 34% demonstrated phenotype drift during limited follow-up. Phenotype distribution did not differ between immunosuppression arms.

Our findings highlight persistent challenges in applying current ISHLT criteria [8, 9]. The most common unclassifiable presentation (isolated FEV_1_ decline with proportional FVC loss maintaining FEV_1_/FVC >0.7) likely represents early BOS before spirometric obstruction becomes apparent. All such re-evaluated cases eventually developed obstruction, supporting this interpretation. The current use of a fixed FEV_1_/FVC threshold (<0.7) rather than trend-based assessment relative to baseline may unnecessarily delay BOS diagnosis. For example, a patient whose FEV_1_/FVC declines from 0.90 to 0.75 exhibits progressive obstruction despite remaining above the 0.7 threshold.

Missing TLC data at EOS is partially due to the fact that EOS was not predictable and phenotyping was not a primary or secondary outcome. It also reflects real-world challenges: patients with advanced CLAD often cannot tolerate body plethysmography due to respiratory distress or low lung volumes. It also reflects study-specific challenges of COVID-related closures of advanced spirometry. Use of FVC decline as a surrogate for restriction, while endorsed for “probable RAS” [5], may overestimate restrictive physiology since obstruction itself can reduce FVC independent of true restriction reduced TLC [9]. This potential bias complicates interpretation, particularly for mixed phenotype classification [10].

The high proportion of unclassifiable cases across multiple cohorts (14–16%) indicates that current definitions do not encompass the full CLAD spectrum. Future ISHLT consensus revisions should consider: trend-based obstruction criteria rather than fixed thresholds, formal recognition of unclassifiable presentations with management guidance, standardised radiological opacity definitions, and clarification of FVC surrogate limitations when TLC is unavailable.

Limitations include use of FVC for restriction when TLC was missing, lack of independent radiological adjudication, small sample sizes precluding phenotype-specific statistical comparisons, and limited follow-up after CLAD diagnosis. The modified Delphi process, while improving agreement through discussion, did not constitute truly independent adjudication after initial voting.

In conclusion, CLAD phenotyping remains challenging despite structured consensus approaches. The substantial rate of unclassifiable cases and phenotype drift, combined with reliance on surrogate criteria when TLC is unavailable, underscore the need for refinement of current classification systems. Prospective studies with protocolised measurements, longer follow-up, and phenotype-specific clinical endpoints are essential to validate the prognostic significance of CLAD phenotypes and inform personalised management strategies.

## Data Availability

Data can be shared if done in compliance with the EU General Data Protection Regulation. The study protocol, statistical analysis plan and monitoring plan will be made available.
